# What to do when the neuropathy worsens after successful heart and liver transplantation in a Glu89Lys Transthyretin Amyloidosis?

**DOI:** 10.1186/1750-1172-10-S1-P37

**Published:** 2015-11-02

**Authors:** Thierry Kuntzer, Francois Ochsner

**Affiliations:** 1 Lausanne University Hospital CHUV, Service of Neurology, Department of Clinical Neurosciences, 1011, Lausanne, Switzerland

## Background

Our patient presented dramatically with a previously unrecognized FAP complicated by heart failure requiring heart transplantation at the age of 49 years and liver transplantation at the age of 51 years. Direct DNA sequencing of the TTR gene showed a heterozygous Glu89Lys mutation in the proband and her daughter (published in Transplantation 2011).

## Methods

Longitudinal follow-up with clinical scores with ancillary testings.

## Results

After her double transplantation, the patient reported over the 8 subsequent years slowly progressive increasing pain and loss of sensation in the feet, slow bowel habit, and delayed urine flow. On examination, we found orthostatic hypotension, sensory loss, muscle weakness, and mild atrophy in the distal lower extremities with reduced Achilles tendon reflexes. Nerve conduction studies revealed a mild decrease of amplitude of motor and absent sensory action potentials and normal velocities except for bilateral slowing within the carpal tunnels. The sympathetic skin response and Sudoscan responses to electrical stimuli was normal in the palms but not in the soles. No complications were seen, such as acute rejection, portal vein thrombosis, or infectious diseases resulting from administration of immunosuppressive drugs.

## Conclusions

As already described, some late-stage patients continue to show FAP progression even after liver transplantation, and longstanding disease is correlated with increased morbidity related to continuing amyloid fibril formation.

